# Paralytic Shellfish Toxins (PST)-Transforming Enzymes: A Review

**DOI:** 10.3390/toxins12050344

**Published:** 2020-05-22

**Authors:** Mariana I. C. Raposo, Maria Teresa S. R. Gomes, Maria João Botelho, Alisa Rudnitskaya

**Affiliations:** 1 CESAM and Chemistry Department, University of Aveiro, 3810-193 Aveiro, Portugal; micr@ua.pt (M.I.C.R.); mtgomes@ua.pt (M.T.S.R.G.); 2 Portuguese Institute for the Sea and Atmosphere, 1449-006 Lisbon, Portugal; mjbotelho@ipma.pt; 3 Interdisciplinary Centre of Marine and Environmental Research, University of Porto, 4050-123 Porto, Portugal

**Keywords:** paralytic shellfish toxins, enzyme, biotransformation, carbamoylase, sulfotransferase

## Abstract

Paralytic shellfish toxins (PSTs) are a group of toxins that cause paralytic shellfish poisoning through blockage of voltage-gated sodium channels. PSTs are produced by prokaryotic freshwater cyanobacteria and eukaryotic marine dinoflagellates. Proliferation of toxic algae species can lead to harmful algal blooms, during which seafood accumulate high levels of PSTs, posing a health threat to consumers. The existence of PST-transforming enzymes was first remarked due to the divergence of PST profiles and concentrations between contaminated bivalves and toxigenic organisms. Later, several enzymes involved in PST transformation, synthesis and elimination have been identified. The knowledge of PST-transforming enzymes is necessary for understanding the processes of toxin accumulation and depuration in mollusk bivalves. Furthermore, PST-transforming enzymes facilitate the obtainment of pure analogues of toxins as in natural sources they are present in a mixture. Pure compounds are of interest for the development of drug candidates and as analytical reference materials. PST-transforming enzymes can also be employed for the development of analytical tools for toxin detection. This review summarizes the PST-transforming enzymes identified so far in living organisms from bacteria to humans, with special emphasis on bivalves, cyanobacteria and dinoflagellates, and discusses enzymes’ biological functions and potential practical applications.

## 1. Introduction

Paralytic shellfish toxins (PSTs) are a group of potent neurotoxins that upon consumption cause paralytic shellfish poisoning (PSP) in fish, birds and mammals including humans. This induced marine toxin disease is characterized by high fatality and a wide distribution around the world [1]. PSTs were initially designated as saxitoxins due to the name of the first identified PST, saxitoxin, isolated from the Alaskan butter clam *Saxidomus giganteus* in 1957 [2]. Since then, more than fifty PSTs differing in structure and toxicity have been reported. All PSTs share a tetrahydropurine ring that can have substitutions at the C11, N1 and C13 positions (R1–R4, Table 1). PST analogues are classified according to an R4 side chain, originating the following main toxin groups in a decreasing order of toxicity: carbamoyl, decarbamoyl and N-sulfocarbamoyl [3,4].

Other rare R4 side chain substituents such as hydroxybenzoate, sulfated-benzoate and acetate have also been identified and structurally described, although the toxicity only of some of them was determined [5,6,7,8]. Physiological actions of PSTs consist of an inhibition of electrical conduction in cells by blocking voltage-gated sodium channels—proteins involved in the nerve signal transduction [9,10]. The affinity of PSTs to the sodium channels is mainly due to the presence of the positively charged guanidinium groups in the tetrahydropurinic ring and hydroxyls at the C12 position [9,10,11]. The PSTs’ affinity is also modulated by the presence of substitutes and is significantly reduced in the presence of sulfate groups at C11 [12].

Specimens from two kingdoms of life are able to produce PSTs: prokaryotic freshwater cyanobacteria, such as *Cylindrospermopsis* spp. and *Lyngbya* spp. [17,18]; and eukaryotic marine dinoflagellates, such as *Gymnodinium catenatum*, *Alexandrium* spp. and *Pyrodinium bahamense* [19,20,21]. Proliferation of toxic algae species can form extensive blooms during which seafood, and especially bivalves, may accumulate high levels of PSTs. Consumption of contaminated bivalves poses a serious threat to human health and harvesting closures lead to economic losses in industries such as aquaculture and tourism. Routine monitoring programs for PSTs in commercial bivalve species have been established in most coastal countries for consumer protection [22].

Awareness of the in vivo PST transformations stemmed from the observed differences in PST profiles and concentrations between contaminated bivalves and PST-producing species as well as between different bivalve species collected from the same area [23,24,25]. This discrepancy has been explained by the differences in uptake, distribution, metabolism and excretion of PSTs by each bivalve species, and by bacterial degradation processes [26,27,28]. Some of the observed in vivo toxin transformations have been attributed to enzymatic action [28] while others have been explained by non-enzymatic reactions, including desulfation, oxidation, reduction and epimerization [24]. Epimerization from the β-epimers to α-epimers by different species of mussels has been widely reported [3,29]. Later, the role of enzymes in the synthesis of PSTs by toxigenic algal cells as well as in the metabolism and elimination of contaminated organisms was recognized.

The knowledge of PST-transforming enzymes is relevant in several aspects. Firstly, it assists in understanding the processes of bivalve toxin accumulation and depuration and consequently in bivalve toxicity. For example, some of the causative dinoflagellates such as *G. catenatum*, produce large quantities of sulfocarbamoyl toxins with very low toxicities, which suffer transformation by some bivalve species to moderately toxic decarbamoyl toxins resulting in significant increase of bivalve toxicity [20,30]. Furthermore, PST-transforming enzymes could also be of interest in the fields of analytical chemistry and pharmacology, as an understanding of enzymatically triggered transformations of PSTs could be useful for the production of individual toxin analogues [31,32]. Such analogues are indispensable for toxicity testing that is needed to establish the toxicity of individual toxins or as a starting point for drug discovery. The potential of compounds such as PST for the development of painkillers due to their affinity to the voltage-gated sodium channels has been highlighted lately [33]. As high systemic toxicity of most naturally occurring PSTs hinder clinical studies, availability of the individual natural analogues with lower toxicity such as N-sulfocarbamoyl toxins or synthetic ones is a prerequisite for therapeutic applications. Individual toxins are also essential as standard reference materials for the development and implementation of toxin detection analytical protocols [32]. PST-transforming enzymes can also be employed for the development of sensors and assays for toxin detection as a less expensive and simpler alternative to liquid chromatography with fluorometric detection (LC-FLD), the reference method in the European Union for PST detection in bivalves [22,34,35]. Enzyme-based biosensors present a less-expensive alternative to the already developed antibody and nerve cell and sodium channel-based assays for PST quantification.

**Figure 1:**
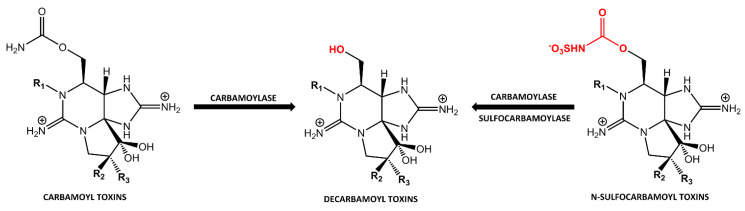
Enzymatic transformations of paralytic shellfish toxins (PSTs) catalyzed by carabamoylase I and sulfocarbamoylase I.

**Figure 2:**
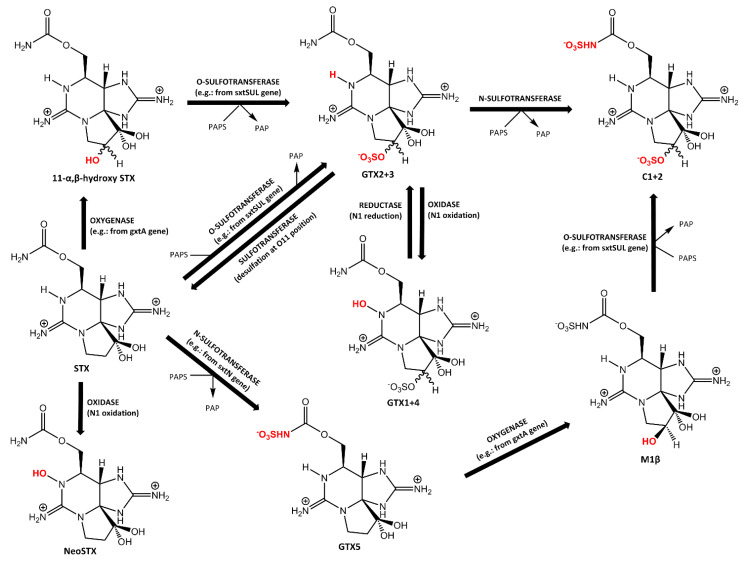
Enzymatic transformations among paralytic shellfish toxins (PSTs) occurring in bivalves, dinoflagellates, fish and humans.

This review summarizes the current knowledge about PST-transforming enzymes, which have been so far identified in bacteria, fish, humans, bivalves and dinoflagellates, with a special emphasis on the latter two. Characteristics of the PST-transforming enzymes, their practical relevance and putative biological functions will be discussed. Enzymes from three families (carbamoylase and sulfocarbamoylase, sulfotransferase and Rieske oxygenase) that were already purified and characterized, will be discussed in Section 2. Other PST transformation reactions, which are also believed to be enzymatically mediated, will be discussed in Section 3. Characteristics of sulfotransferases isolated from different dinoflagellate species are summarized in Table 2 and a summary of PST-transforming enzymes and the organisms in which they have been identified is presented in Table 3. The main enzymatic reactions involving transformation of PSTs are schematically represented in Figure 1 and Figure 2. Earlier studies on enzymatic PST biotransformation in bivalves were summarized in [28].

## 2. Characterized PST-Transforming Enzymes

### 2.1. Carbamoylase and Sulfocarbamoylase

Discrepancies between PST profiles in contaminated bivalve species and causative phytoplankton, reported since the late 70s [36,37], were hypothesized to arise from selective uptake or excretion of specific toxins by bivalves and enzymatic and/or chemical transformations. The enzymatic nature of some of those PST transformations was first confirmed in 1983 by Sullivan et al., who described decarbamoylation of carbamoyl and N-sulfocarbamoyl toxins in crude extracts of little-necked clam *Protothaca staminea* [25] (Figure 1). Inactivation of this reaction by heat, an addition of organic solvent (methanol) or a low pH provided evidence that decarbamoylation is enzymatically catalyzed. It was found that N-sulfocarbamoyl toxins were more rapidly converted than the carbamoyl toxins and that reactions proceeded faster in homogenates of the littleneck clam viscera compared to other analyzed tissues (mantle, muscle and siphon). No toxin transformations were observed in either mussels (*Mytilus edulis*) or butter clams (*S. giganteus*). Later, these findings were corroborated by Fast et al. [24] and Buzy et al. [38].

Oshima showed the presence of a similar toxin transformation in the clams *Mactra chinensis* and *Peronidia venulosa* [3]. While *M. chinensis* hydrolyzed both N-sulfocarbamoyl and carbamoyl toxins, *P. venulosa* did not hydrolyzed the latter group. Even though decarbamoylation of toxins could also take place in the presence of natural reductants such as cysteine and glutathione, this alternative reaction would require more severe conditions and the reaction rate would be much lower, leading to the conclusion that toxin conversion observed in bivalve tissues was catalyzed by enzymes [3]. Based on the results obtained from the bivalve extracts, the same research group successfully proceeded to extract, purifify and characterize those enzymes responsible for the observed PST transformations.

An enzyme named carbamoylase I was firstly extracted from the clam *M. chinensis* [39]. Carbamoylase I was found to be a 190 kDa glycoprotein with two subunits bound through S–S linkage. The reaction mechanism was confirmed using incubation of purified enzymes with individual PSTs. It consisted of a cleavage of carbamoyl or sulfocarbamoyl moiety of carbamoyl and N-sulfocarbamoyl toxins, respectively, without any other structural modifications (Figure 1). The presence or absence of a hydroxyl moiety at the N1 position of the substrate toxins did not significantly alter the reaction rate, while stereochemistry of the substitutes at C11 did. The consumption rate was found to be lower for the toxins in the sulfate group as well as α-epimers at the C11 position. For α- and β-epimers, the consumption rate could differ by as much as 16.5 times [39]. While all tissues of *M. chinensis* were found to have at least some enzymatic activity, both specific activities and the activity of crude extracts were highest in the digestive gland and crystalline style.

An enzyme extracted from another clam species, *P. venulosa*, was named sulfocarbamoylase I, as it hydrolyzed only the sulfated carbamoyl group of N-sulfocarbamoyl PSTs [40] (Figure 1). Sulfocarbamoylase I was found to be a protein of 300 kDa comprising two ionically bound subunits of 150 kDa each. Though enzymatic activity was more evenly distributed in the tissues of *P. venulosa* compared to *M. chinensis*, the highest enzymatic activity in *P. venulosa* was also found in the digestive gland and crystalline style. Higher biotransformation activity associated with the digestive system was reported for several other bivalve species [24,41,42,43]. This observation is understandable taking into account that this is the site where the liberation of PSTs from ingested dinoflagellates take place and where enzymes involved in the digestive metabolism are present. Similar to carbamoylase I, sulfocarbamoylase I has serine residues in its catalytic site and both showed the maximum activity at pH 6.8, while optimal temperature was slightly different—20 °C for the former and 25 °C for the latter. Another similarity between the two enzymes is the higher specificity to β-epimers compared to the α-epimers at C11 position, as demonstrated by the higher affinity of sulfocarbamoylase I to C2 compared to C1 and the higher affinity of carbamoylase I to GTX3 and GTX4 compared to GTX2 and GTX1. However, the enzymes exhibited discordant specificity to GTX5 and GTX6, which differ by the presence of OH group at N1, with carbamoylase I having a higher consumption rate of GTX5 and sufocarbamoylase I of GTX6. In the case of sulfocarbamoylase I, the sulfonyl moiety in the carbamoyl side chain of substrates is essential for substrate recognition as this enzyme only hydrolyzes N-sulfocarbamoyl toxins.

Apart from *M. chinensis* and *P. venulosa*, decarbamoylation of PSTs attributable to enzymatic action was observed in several other bivalve species, though no enzyme extraction and purification was reported. Specificity of enzymatic reactions vary between species, yet they all share common features such as a reaction mechanism, i.e., cleavage of the carbamoyl or sulfocarbamoyl moiety without other structural changes, a higher specificity towards β-epimers at the C11 position than to the α-epimer and higher enzymatic activity in the digestive gland compared to the other tissues. Bivalve species (other than *M. chinensis* and *P. venulosa*) possessing carbamoylase activity are summarized below.

The appearance of decarbamoyl PST derivatives in clam *Ruditapes decussatus* fed with *G. catenatum* cells containing predominantly N-sulfocarbamoyl toxins has been described in [44]. The decarbamoylation of N-sulfocarbamoyl and carbamoyl toxins has also been observed in surf clam *Spisula solida*, which were naturally and artificially contaminated by *G. catenatum*, as well as in their digestive gland homogenates incubated with toxins [23,32,41,45]. Inactivation of the toxins’ transformations by heating the tissue homogenates confirmed the enzymatic nature of the reaction [41]. Decarbamoylation was also reported in the clam species *Spisula solidissima* [46], *Paphies donacina* [47] and *Paphies subtriangulata* [48], geoduck clam *Panopea globosa* [43], short-necked clam *Tapes japonica* [49,50] as well as in the aforementioned Pacific littleneck clam *P. staminea* [24,25,38]. Lower enzymatic activity was reported for sea scallop *Placopecten magellanicus* [26,46], quahog *Mercenaria mercenaria* [51], cockle *Cerastoderma edule* [52], peppery furrow shell *Scrobicularia plana* [41] and soft-shell clam *Mya arenaria* [24]. Contrary to the fast decarbamoylation by the digestive gland of *S. solida* clam, which transformed 95% of carbamoyl and N-sulfocarbamoyl toxins after 24 h of incubation, enzymatic activity of the digestive gland of *S. plana* was lower, converting 5% and 41% of PST after 24 h and 6 days of exposure, respectively [41]. Though de novo appearance of dcSTX was observed in the naturally contaminated *C. edule*, it did not occur after in vitro incubation of bivalve homogenate with STX for 24 h, indicating that enzymatic activity of *C. edule* is even lower than that of *S. plana*. Similarly moderate enzymatic activity was reported for the *Solen grandis*, *Panope japonica* and *Patinopecten yessoensis*, which converted ca. 30%–35% of GTX1+4 toxins after 24 h of incubation [39]. Enzymatic conversion of PSTs was also reported for the Asian green mussel *Perna viridis* and scallop *Chlamys nobilis* [53].

The pronounced effect that orientation of the sulfate group at the C11 position has on enzyme affinity, previously seen for *M. chinensis* and *P. venulosa,* was confirmed in other bivalves species by Fast et al. [24] Decarbamoylation of the C2 (β-epimer) toxin occurred faster than the C1 (α-epimer) toxin in tissue homogenates of *P. staminea.* In contrast to the *M. chinensis* carbamoylase, the enzyme present in *P. staminea* and *S. solida* was affected by the presence of a hydroxyl moiety at the N-1 position, which diminished the reaction rate, since transformation of N-hydroxylated toxins such as e.g., GTX1+4, were less complete when compared to other PSTs [24,32].

Enzymatic activity consistent with the presence of carbamoylase is relatively uncommon among bivalves and has been demonstrated only for some clam species while most bivalves either do not have it at all or have it at very low levels. Comparison of eighteen species of Japanese bivalve mollusks has shown that only *M. chinensis* possesses carbamoylase activity [3]. Moreover, after the in vitro incubation with STX standard, the formation of dcSTX in *M. edulis*, *C. edule*, *Crassostrea gigas* and *Donax trunculus* was not observed [41]. Even in specimens from the same genus, carbamoylase activity was not always detected: although *Mactra stultorum* belongs to the same genus as *M. chinensis*, no enzymatic conversion was observed in the former [42]. Only one study, which exclusively targeted recently described benzoate PSTs reported a widespread carbamoylase activity in bivalves [54]. In vitro conversion of benzoate toxins into dcGTX2+3 and dcSTX was observed after incubation with the digestive gland homogenate of blue mussels (*M. galloprovincialis*), common cockles (*C. edule*), clams (*R. decussatus*, *Venerupis pullastra* and *S. plana*), oysters (*Crassostrea japonica*) and offshore clams (*D. trunculus, S. solida* and *Chamelea gallina*). Conversion of benzoate toxins was attributed to the enzymatic activity as it was partly heat-inactivated [54].

Whilst carbamoylase is present in several bivalve species, sulfocarbamoylase appears to be rarer and was unambiguously identified only in *P. venulosa* [40]. However, preferential decarbamoylation of N-sulfocarbamoyl toxins was reported for UK surf clam *S. solida* [32] and soft-shell clam *M. arenaria* [24]. Complete conversion of GTX5 and C1+2 toxins was observed in *S. solida* homogenates, while transformation of STX and GTX2+3 was less complete (80%–95%) and transformation for the N-hydroxylated toxins GTX1+4 and NEO was even lower [32]. Interestingly, substrate specificity of enzymes from the same species collected in different geographical areas varied as was observed for *S. solida* in UK Portuguese Atlantic coastal zones [32,41]. *M. arenaria* that have limited capacity to transform toxins such as C2 and GTX1+4 was found incapable of converting C1, GTX2+3, NEO and STX [24]. Enzymatic activity of tissues of *P. viridis* and *C. nobilis* was studied in the bivalves contaminated by feeding with cultured *Alexandrium tamarense* that produce predominantly C2 toxins [53]. Therefore, it is not clear if the enzyme present in these mollusks is capable of converting carbamoyl toxins as well, i.e., if it is closer in properties to carbamoylase or sulfocarbamoylase.

The ability to decarbamoylate PSTs is not restricted to bivalves and has been observed in other organisms. Gram-negative, rod-shaped bacteria isolated from bivalves have been found capable of metabolizing PSTs. One particular isolate from mussels (*M. edulis*) was demonstrated to convert carbamoyl toxins, GTX2+3, in the decarbamoylated dcGTX2+3 [55]. Evidence of PSTs’ decarbamoylation in humans was obtained through an analysis of tissues and bodily fluids of mortal victims of paralytic shellfish poisoning [56]. The appearance of small amounts of dcSTX was observed in liver, kidney and lung samples while this toxin was absent in gastric content and consumed bivalves. This was interpreted as enzymatic decarbamoylation of STX, which was the major toxin present in the gastric content, in metabolically active tissues. No decarbamoylation of gonyautoxins has been observed, which may be due to significantly lower (approximately ten-fold) concentrations of these toxins compared to STX or a higher affinity of human carbamoylase to STX.

### 2.2. Sulfotransferases

#### 2.2.1. Sulfotransferases Involved in PST Biosynthesis

The sulfonation of biomolecules catalyzed by enzymes-sulfotransferases has long been known to take place in a variety of organisms, from prokaryotes to multicellular species. Sulfonation is an important pathway in metabolism of xeno- and endobiotics and drugs as it increases the water solubility of molecules and decreases their biological activity. In relation to PSTs, sulfotransferases were shown to be involved in the synthesis of sulfated analogues, detoxification and other metabolic transformations.

Sulfotransferase involved in the synthesis of sulfated PSTs in dinoflagellates was first reported by Oshima in 1995 [3]. Enzymatic conversion of GTX2+3 into C1+2 was observed in extracts of both toxic and nontoxic isolates of *G. catenatum* upon the addition of 3′-phosphate-5′-phosphosulfate (PAPS), which served as a sulfonate donor (Figure 2 and Table 2).

Furthermore, several sulfotransferases were extracted from dinoflagellates. N-sulfotransferase (N-ST) purified from *G. catenatum*, was shown to transfer sulfate group from PAPS to N21 atom of hydropurine ring of GTX2+3 and STX producing C1+2 and GTX5, respectively. Higher affinity to STX than to GTX2+3 was found [57]. Absense of hydroxyl at N1 plays an important role in substrate specificity of the enzyme since N-ST was not active toward NeoSTX and GTX1+4 toxins, which are hydroxylated at N1. N-ST was found to be a monomer with a molecular mass of 59 kDa, having an optimal activity at pH 6 and 25 °C, which was enhanced by Mg^2+^ and Co^2+^ [57,58].

An N-ST with very similar properties was extracted and partially purified from dinoflagellate *A. catenella* [59]. *A. catenella* N-ST did not require divalent cations and had lower optimal activity temperature—15 °C vs. 25 °C—compared to *G. catenatum* N-ST. Although initially N-ST was not detected in *A. tamarense* [3], a later study demonstrated its presence in crude extract of this dinoflagellate. The *A. tamarense* N-ST was not active towards STX, but only towards GTX2+3 [60]. These results suggested that the properties and functions of N-ST are species-specific.

Another type of sulfotransferase, O-sulfotransferase (O-ST), was purified from the cytosolic fraction of the toxic *G. catenatum*. The O-ST transfers the sulfate group of PAPS only to O22 position of 11-α,β-hydroxy STX producing GTX2+3 (Figure 2 and Table 2) [61]. The enzymatic activity concordant with the O-ST mode of action has been detected earlier in this dinoflagellate [57,58].

Association of sulfotransferase presence with sulfated PST synthesis in dinoflagellates was further corroborated by the proteomic analysis of differentially expressed proteins in toxic *A. catenella* that was harvested at different toxin biosynthesis stages (non-toxin synthesis, initial toxin synthesis and toxin synthesis) coupled with the cell cycle [62]. Among differentially expressed proteins, nine were associated with sulfated PST biosynthesis including putative sulfotransferase.

As sulfotransferases are involved in the biosynthesis of PSTs, their presence can be expected in other paralytic toxin-producing organisms such as cyanobacteria. Gene sequencing of toxic cyanobacteria species permitted to identify PST biosynthesis gene clusters in which genes encoding two sulfotransferases—N-sulfotransferase (sxtN) and O-sulfotransferase (sxtSUL)—were found [63]. Based on gene sequences, gene expression levels and toxins produced by organisms, it was inferred that sulfotransferase encoded by the sxtN gene incorporates a sulfonyl group at the N21 position of the carbamoyl group, yielding e.g., GTX5 from STX, while sulfotransferase encoded by by sxtSUL gene transfered the sulfate group to the C11 position, thereby rendering GTX2+3 from STX or dcGTX2+3 from dcSTX. The joint action of O- and N-sulfotransferases would be necessary to produce C1+2 toxins [63,64]. Genes encoding both sulfotransferases were identified in *Cylindrospermopsis raciborskii* T3 [63,65], *Raphidiopsis brookii* D9 [63], *Anabaena circinalis* and *Aphanizomenon sp. Nostocales* [66,67], *Scytonema crispum* [68] and *Microseira wollei* (formerly *Lyngbya wollei*) [64].

Apart from purification from natural sources, expression can be used for obtaining pure enzymes and unambiguously determining their function and substrate preferences. Confirmation of the function of expressed sulfotransferases and of PST biosynthesis pathways have been reported in [69]. O-sulfotransferase encoded by the sxtSUL gene from *M. wollei* and the N-sulfotransferase encoded by the sxtN gene from *Aphanizomenon sp. NH-5* were expressed, purified and characterized with respect to their activity on a range of substrates. STX was found to be the preferential substrate for N-sulfotransferase, which converted it to a single product, GTX5. Oxygenase (GtxA), which will be discussed in Section 2.3, oxidized both STX and GTX5 at the C11 position converting them to 11-β-hydroxySTX and M1β, respectively. SxtSUL converted 11-β-hydroxySTX to GTX3, which partly epimerized to GTX2 over time, and M1β to C2. Thus, cascade reactions involving sxtN and sxtSUL sulfotransferases and STX as a substrate was allowed to obtain a mixture of GTX5, GTX3, GTX2 and C2 toxins (Figure 2). These results confirmed with some alterations the PST biosynthetic pathway proposed by Yoshida et al. [61] Their work also confirmed the formation of 11-β-hydroxySTX from STX as a precursor of gonyautoxins in PST synthesis, which was first hypothesized as a possible intermediate compound [61] and later detected in mussels collected during *A. tamarense* toxic bloom [70].

#### 2.2.2. Sulfotransferases Involved in PST Metabolism

Another type of PST transformation involving cleavage of sulfate group at the C11 position has been described for several bivalves exposed to toxins. Though initially this reaction was deemed to be a redox process occurring in the presence of glutathione [37], it was later attributed to the action of sulfotransferase. In the reaction of desulfation of PSTs at C11, sulfated toxins serve as a donor of the sulfate group, which is transferred to various acceptor molecules, endogenous metabolites, or xenobiotics. Desulfation at the C11 position resulting in the conversion of GTX2+3 toxins to STX and GTX1+4 to NeoSTX was observed in the mussel *M. edulis* [29], scallop *P. magellanicus* [3,37], geoduck clam *P. globosa* [43] and scallop *Chlamys farreri* [71].

Desulfation of PSTs can also give rise to the hydroxyl analogues M1–M10, which possess the OH group at the C11 position. The appearance de novo of hydroxylated M-toxins in the PST contaminated shellfish, which were absent in toxin-producing organisms, was reported for scallop *C. farreri* and mussel *M. galloprovincialis* [72,73], and for scallop *P. yessoensis* and clam *Saxidomus purpuratus* [74]. It was hypothesized that M1 is converted from C1+2, M7 from C3+4, M2 from GTX2+3 and M8 from GTX1+4. Newly formed toxins M1, M2, M7 and M8 further suffered oxidation at C11, leading to the formation of oxyhydroxide analogues M3, M4, M9 and M10, respectively [72].

Bacteria present in marine animals are also capable of PST transformation as was demonstrated for *Pseudomonas* sp. and *Vibrio* sp. isolated from crab, *Atergatis floridus,* and from turban shell, *Turbo argyrostoma*, which converted GTX2+3 to STX [75,76]. Inhibition of the reaction in the presence of toluene confirmed its enzymatic nature. It was also shown that the reductive elimination is accomplished by bacteria rather than by marine invertebrate enzymes once the reaction did not proceed in the extracts obtained under bacteriostatic conditions.

Similar to other enzymatic PST transformations in bivalves, desulfation results in the formation of significantly more toxic compounds. Though the physiological role of such PST transformations remains unclear, a recent study combining analysis of genome, transcriptome, proteome and PST profiles provided a new insight into its purpose [71]. PST quantification in the tissues of the scallop *C. farreri* fed with toxic dinoflagellate *A. minutum* revealed sulfotransferase activity in the mollusk kidneys. The appearance of the STX, absent in the algae toxin profile, together with the enrichment of the cytosolic sulfotransferase genes in the kidneys indicated the enzymatically mediated desulfation of donor molecules such as GTXs. Thus, sulfotransferase in scallop kidneys cleaves the sulfate group from the C11 position of GTX2+3, which are abundant in *A. minutum*, producing STX. Furthermore, mutations conferring an increased resistance to PST and tetrodotoxins have been found in two sodium channel genes of *C. farreri*. Presence of these mutations together with the downregulation of sodium channel gene expression in toxin-rich organs of the scallop explain bivalve capability to tolerate neurotoxins [77,78]. These results also suggest that scallop uses hepatopancreas to accumulate neurotoxins and kidney to transform them into highly toxic forms through sulfotransferases, probably as a deterrence against predation, while it achieves neurotoxin resistance through point mutations in sodium channels and downregulation of their expression in toxin-rich organs [71].

### 2.3. Rieske Oxygenase

Rieske oxygenases belong to the group of non-heme iron dependent oxidative enzymes that catalyze a wide range of reactions of xenobiotics’ biodegradation and biosynthesis of bioactive compounds [79]. Recently, Rieske oxygenases capable of carrying C–H bond oxygenation reactions were identified among enzymes in the putative STX synthesis gene clusters of cyanobacteria *M. wollei* (formerly *L. wollei*) [64]. Three oxygenases, which were expressed and purified, were found to be involved in the biosynthesis of PST precursors and intermediates. One of them, encoded by GxtA, was found to react with toxins, hydroxylating β C–H bond at the C11 position of STX, dcSTX, NeoSTX and GTX5 producing the respective 11-β hydroxylated compounds, which upon action of sulfotransferases give origin to the sulfated gonyautoxins and N-sulfocarbamoyl toxins (Figure 2) [69]. Though oxygenase encoded by GxtA hydroxylates several substrates, it maintains an exquisite selectivity for the β C–H bond at the C11 position. Another oxygenase, encoded by StxT, hydroxylates β-saxitoxinol at C12 giving rise to saxitoxin.

## 3. Other PST-Transforming Enzymes

### 3.1. Reduction at N1 Position

Enzymatic reduction at the N1 position of PSTs was described in one of the first reports of enzymatic activity in bivalves [37]. The foot and adductor muscles of *P. magellanicus* scallop were shown to rapidly convert GTX1+4 and NeoSTX to STX, which was ascribed to the enzymatic hydrolysis of the OH group at the N1 position and consequent hydrolysis of the sulfate group at C11 by sulfotransferase in the case of gonyautoxins.

Conversion of GTX1+4 to the corresponding N1-deoxygenated analogues GTX2+3 was observed in the viscera homogenates of surf clam *Pseudocardium sachalinensis* [80], purple clam *Hiatula rostrata* [81] and Chinese scallop, *C. farreri* [82]. Loss of PST-transforming activity after heating and its significant decrease at low pH permits the conclusion that this reduction is enzymatically mediated. Use of bacteriostatic conditions for enzymatic reactions also allowed the discardation of the possible contribution of bacteria to the observed toxin conversions [80].

For their part, bacteria isolated from dinoflagellates, bivalves and other marine invertebrates also catalyze N1 reduction of PSTs. N1 reduction of GTX1+4 producing GTX2+3 was observed in the cultures of α- and γ-Proteobacteria isolated from toxic (*A. tamarense* and *A. lusitanicum*) and nontoxic (*Scrippsiella trochoidea*) dinoflagellates [83], *Pseudomonas* sp. and *Vibrio* sp. isolated from the viscera of crab, *A. floridus* and the turban shell, *T. argyrostoma* [76], and in the bacteria isolates from mussels (*M. edulis*) and razor fish (*Ensis arcuatus*) [55]. Bacteria isolates from crab and turban shell proceeded to convert the produced GTX2+3 into STX. The rapid increase of GTX2+3 and the delayed appearance of STX in the medium led to the conclusion that the reductive elimination of OH at N1 precedes desulfation at C11.

Only a few of several bacterial isolates that were obtained from mussels and razor fish displayed PST-transforming activity [55]. Endogenous metabolism of most of the tested bacterial isolates was inhibited in the presence of PST. It was hypothesized that PST-transforming bacteria use toxins as a carbon or energy source. Once degradative activity of isolates capable of transforming PSTs was inhibited by the high toxin concentrations or abundance of nutrients (when grown in nutrient-rich marine broth), it was proposed that marine bacteria would only transform PSTs when other carbon sources have been exhausted, as observed for the degradation by the aquatic bacteria of the cyanobacterial toxin microcystin [55].

### 3.2. Oxidation at the N1 Position

The presence of an oxidase activity in dinoflagellates was initially demonstrated in *A. tamarense* extract, in which transformation of GTX2+3 into GTX1+4 requiring hydroxylation at the N1 position has been observed (Figure 2) [3]. Similar oxidase activity was reported in bacteria [83]. Among PST-transforming bacteria isolated from toxigenic *A. lusitanicum* and non-toxic *S. trochoidea* dinoflagellates, two isolates identified as α-Proteobacteria belonging to *Ahrensia* sp. and *Caulobacter* sp. were capable of hydroxylating GTX2+3, producing GTX1+4. Interestingly, bacteria isolated from the non-toxic strain of *A. tamarense* did not display PST-transforming activity.

Sequencing of PST gene clusters in toxigenic cyanobacteria species *C. raciborskii T3*, *A. flosaquae NH-5* and *M. wollei* identified a gene that encodes an enzyme that catalyzes the hydroxylation at the N1 position, as, for example, the conversion of STX into NeoSTX [64,65]. This finding was further confirmed by the absence of this gene in any of the analyzed strains of *A. circinalis* species, which does not produce N1-hydroxylated PSTs.

The evidence of N1 hydroxylation in the human body was provided by the analysis of tissue and body fluid samples obtained from the mortal victims of paralytic shellfish poisoning in 2002 [56]. The major toxin detected in the victims’ gastric content was STX, while gonyautoxins GTX1+4, GTX2+3 and GTX5 were detected at lower concentrations. This toxin profile was in line with the average profile reported earlier for filter-feeding mollusks from the Chilean coast, which were the cause of poisoning [84]. An increase of GTX1+4 concentrations and the appearance of NeoSTX concomitantly with a decrease of GTX2+3 and STX concentrations were detected in several organ tissues and bodily fluids, with the most pronounced differences found in urine and bile. This transformation was hypothesized as an enzymatic oxidation at the N1 position of the tetrahydropurine ring belonging to phase I metabolic reactions, which functionalize xenobiotics prior to the next step of enzymatic detoxication. In vitro incubation of GTX2+3 toxins with microsomal fractions isolated from healthy human liver demonstrated formation of GTX1+4 epimers, confirming enzymatic oxidation at the N1 position [85,86].

### 3.3. Enzymes Involved in PST Detoxification

Enzymatic reactions play an important role in the detoxification of xenobiotics by transforming them into more polar and hydrophilic compounds, which facilitates their excretion. Two enzymes belonging to the phase II conjugation of enzymes of detoxification, glutathione S-transferase (GST) and glucuronosyltransferases, were described to convert PSTs in the susceptible organisms that were exposed to PSTs. Though these enzymes are not specific to PSTs and act on several xenobiotics apart from toxins, a short description of their action on PST was included in this review.

Glutathione S-transferase (GST) catalyzes the conjugation of xenobiotics with glutathione, promoting the excretion of those compounds [87]. GST induction in the liver following intracoelomical injection of a mixture of PSTs extracted from toxic dinoflagellate has been demonstrated in Atlantic salmon (*Salmo salar*) [88] and white seabream (*Diplodus sargus*) [89]. Interestingly, while exposure to carbamoyl and N-sulfocarbamoyl PSTs led to the increase of GST activity, exposure to the more lipophilic hydroxybenzoate PSTs led to the short-term depletion of GST [90] (Figure 2). Thus, it was hypothesized that the exposure to hydroxybenzoate PST analogues impair the protective action of GST, increasing fish susceptibility to other PSTs.

Uridine diphosphate glucuronosyltransferases (UGTs) catalyze a glucuronidation reaction that involves the transfer of glucuronic acid to relatively non-polar substrates, producing hydrophilic conjugates in order to make them easier to excrete [91]. A glucuronidation reaction at C12-OH of the tetrahydropurine ring was observed in vitro in human liver microsomes [85,86]. Incubation of liver microsomes with GTX2+3, STX and NeoSTX and with co-factors uridine diphosphate, glucuronic acid and nicotinamide adenine dinucleotide phosphate yielded glucuronated metabolites of these toxins with 85% of toxins converted within 5 h. Higher affinity of UGTs to gonyautoxins GTX2+3 compared to STX and NeoSTX was observed.

## 4. Concluding Remarks

This review summarizes the current knowledge of enzymes involved in the metabolism of PSTs. There are still some gaps in this field due to the difficulties in studying the processes and metabolic pathways involved in the transformation of toxins. Results of in vivo experiments carry uncertainty related to the translocation of toxins between tissue compartments, and their differential retention and elimination. On the other hand, in vitro studies may exclude the interference of physiological processes that might affect the biotransformation. Characterization of individual enzymes has been hampered by their low stability and low concentrations at which they are present in living organisms, making their purification laborious. Recently, significant progress in the characterization of PST-transforming enzymes and elucidation of PSTs’ biosynthesis pathways have been achieved by recombinant protein expression [31,69].

Yet an understanding of the biotransformation of PSTs as well as the enzymes that catalyze those transformations is of critical importance. Biotransformation of PSTs taking place in some bivalve species may lead to the formation of highly toxic compounds from less toxic analogues produced by the bloom causative phytoplankton, such as, for example, the formation of decarbamoyl toxins from gonyautoxins. Sometimes biotransformations can lead to the appearance of new PSTs that cannot be biosynthesized by PST-producing organisms. Therefore, a knowledge of PST biotransformation is of paramount importance for the prediction of the toxicity of different bivalve species during dinoflagellate blooms, permitting more efficient risk management.

Another area which can benefit from a knowledge of PST-transforming enzymes, is the preparation of certified reference materials. PSTs are a group of more than 50 analogues, several of which are encountered simultaneously. The move away from mouse bioassays in monitoring programs means that individual toxins need to be identified and quantified using an official reference method such as liquid chromatography with fluorescence detection [22], after which the toxicity estimate is calculated using toxicity factors established for individual toxins [4]. Evidently, individual toxins are indispensable for toxicity testing aimed at establishing their toxicity. Furthermore, appropriate contaminated bivalve matrices are required for method validation and quality control. Such reference materials are usually produced by spiking nontoxic bivalves with toxic dinoflagellates, or through bivalve feeding with toxic algae in laboratory conditions. However, issues with the instability of such reference materials are well known [92]. This process can be significantly aided by the use of PSTs transforming enzymes and allowing the targeted production of reference materials which include a wider range of toxins than normally present in naturally incurred bivalves [32].

PSTs’ physiological actions consist of blocking the outer pore of the voltage-gated sodium channel, impeding nerve impulse propagation at the neuromuscular junction and causing paralysis [93]. The same property makes them interesting candidates for the development of anesthetics and muscle relaxants with potential applications in the treatment of neurodegenerative diseases and pain [31,33,69]. Importantly, these compounds elicit the desired analgesic effect without the possible cardiotoxicity and abuse potential observed with conventional local anesthetics. Several trials of gonyautoxins GTX2+3 injections as local anesthetics in humans [94,95,96] and NeoSTX in piglets [97] have been carried out with promising outcomes. However, the high toxicity of these compounds result in very narrow windows between effective and toxic concentrations, making their therapeutic applications challenging. An approach to the development of the molecules with enhanced therapeutic potential may start with less toxic PST analogues. It is known that sodium-channel affinity of STX decreases significantly with oxidation at N1 (NeoSTX) or sulfation at C11 (gonyautoxins). PST-transforming enzymes offer the possibility of targeted toxin modifications for producing a range of STX analogues with low toxicity including non-natural ones for evaluation as candidate therapeutics.

Last but not least, PST-transforming enzymes may find applications in the development of analytical tools for toxin detection. Interest in sensors and assays for the fast screening of PSTs has led to the development of several antibody-based ELISA kits and biosensors [98,99] and assays based on sodium channels and neural cells [100,101,102]. However, these antibody-based kits require an animal host for their production and the sodium channel and neural cell assays involve laborious preparations and use of radioisotopes. Enzymes may serve as an alternative to the development of the fast and low-cost analytical tools for PST detection. Since the first enzymatic electrode was proposed 40 years ago [103], enzyme-based biosensors have received significant attention due to the advantages of the association of the biocatalytic activity of enzymes with the lower costs and reusability of sensors [104,105,106,107]. As a first step in this direction, the first carbamoylase-based assay with potentiometric detection for the quantification of GTX5 was developed [108].

Overall, this review has gathered information on the main PST-transforming enzymes found among different organisms, since they all can have an importance in several of the fields discussed above. Despite significant progress, further research is necessary in order to improve our knowledge of PST-transforming enzymes.

## Figures and Tables

**Table 1:** Structure of paralytic shellfish toxins and correspondent TEF (toxicity equivalency factor) [4], toxicity obtained by mouse bioassay (MBA) relative to STX [6,13,14] or dissociation constant K_D_ characterizing the potency of binding to rat brain sodium channels K_D_ (nM) [15,16] for compounds for which TEF is not defined. STX—saxitoxin, GTX—gonyautoxin.

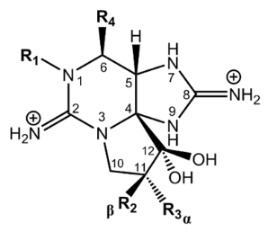				Basic Structure				
**Group**	**Toxin**	**R1**	**R2**	**R3**	**R4**	**TEF**	**MBA**	**K_D_**
Carbamoyl	STX	H	H	H	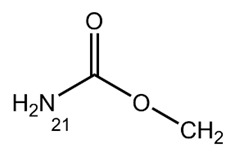	1.0	1.0	0.5
	NeoSTX	OH	H	H		1.0		
	GTX1	OH	H	OSO^−3^		1.0		
	GTX2	H	H	OSO^−3^		0.4		
	GTX3	H	OSO^−3^	H		0.6		
	GTX4	OH	OSO^−3^	H		0.7		
	M2	H	OH	H		nk		
	M4	H	OH	OH				
Decarbamoyl (dc)	dcSTX	H	H	H	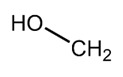	1.0		
	dcNeoSTX	OH	H	H		0.4		
	dcGTX1	OH	H	OSO^−3^			0.5	
	dcGTX2	H	H	OSO^−3^		0.2		
	dcGTX3	H	OSO^−3^	H		0.4		
	dcGTX4	OH	OSO^−3^	H			0.5	
	▪ LWTX4	H	H	H			0.004	
N-sulfocarbamoyl	GTX5 (B1)	H	H	H	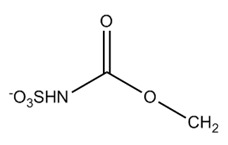	0.1		
	GTX6 (B2)	OH	H	H		0.1		
	C1	H	H	OSO^−3^			0.02	
	C2	H	OSO^−3^	H		0.1		
	C3	OH	H	OSO^−3^			0.01	
	C4	OH	OSO^−3^	H		0.1		
	M1	H	OH	H		nk		
	M3	H	OH	OH				
Mono-hydroxybenzoate	GC1	H	H	OSO^−3^	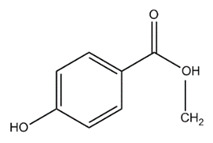			3.4–4.4
	GC2	H	OSO^−3^	H				3.4–4.4
	GC3	H	H	H				2.2
	*GC4	OH	H	OSO^−3^		nk		
	*GC5	OH	OSO^−3^	H				
	*GC6	OH	H	H				
Di-hydroxybenzoate	#GC1a	H	H	OSO^−3^	Di-hydroxy-benzoate analogue	nk		
	#GC2a	H	OSO^−3^	H				
	#GC3a	H	H	H				
	#GC4a	OH	H	OSO^−3^				
	#GC5a	OH	OSO^−3^	H				
	#GC6a	OH	H	H				
Sulfated-benzoate	#GC1b	H	H	OSO^−3^	Sulfated-benzoate-analogue	nk		
	#GC2b	H	OSO^−3^	H				
	#GC3b	H	H	H				
	#GC4b	OH	H	OSO^−3^				
	#GC5b	OH	OSO^−3^	H				
	#GC6b	OH	H	H				
Deoxydecarbamoyl (do)	doSTX	H	H	H	CH_3_	nk		
	doGTX2	H	H	OSO^−3^				
	doGTX3	H	OSO^−3^	H				
Acetate	▪ LWTX1	H	H	OSO^−3^	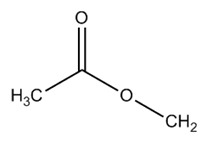		0.07	
	LWTX2	H	H	OSO^−3^			0.004	
	LWTX3	H	OSO^−3^	H			0.02	
	LWTX5	H	H	H			0.14	
	▪ LWTX6	H	H	H			0.004	

* Not structurally characterized; ^#^ R4 group not structurally characterized; ▪12-deoxy compounds; nk = not known.

**Table 2:** Characteristics of sulfotransferases (N-ST and O-ST) from several dinoflagellate species.

ST Type	N-ST			O-ST
Dinoflagellate species	*G. catenatum*	*A. catenella*	*A. tamarense*	*G. catenatum*
Optimal activity pH	6	6	6	6
Optimal activity temperature	25 °C	15 °C	15 °C	35 °C
Molecular mass	59 kDa	60 kDa	nk	65 kDa
Structure	monomeric	nk	nk	monomeric
Cation requirement	Mg^2+^, Co^2+^ (enhanced effects)	none	none	none
Sulfate Donor	PAPS	PAPS	PAPS, DMSO, MgSO_4_	PAPS
Reactions	GTX2+3 → C1+2 STX → GTX5	GTX2+3 → C1+2 STX → GTX5	GTX2+3 → C1+2	11-α,β-hydroxy STX → GTX2+3

nk—not known, PAPS—3′-phosphate-5′-phosphosulfate.

**Table 3:** Enzymes or enzymatic-mediated transformations present in different species.

Enzyme/Enzymatic Mediated Transformations	Organism	References
Carbamoylase	*Mactra chinensis*	[3,39]
	*Protothaca staminea*	[24,25,38]
	*Spisula solidissima*	[46]
	*Spisula solida*	[23,32,41,54] *
	*Ruditapes decussatus*	[44,54] *
	*Scrobicularia plana*	[41,54] *
	*Paphies donacina*	[47]
	*Paphies subtriangulata*	[48]
	*Panopea globosa*	[43]
	*Tapes japonica*	[46,47]
	*Plactopecten magellanicus*	[26,46]
	*Mercenaria mercenaria*	[51]
	*Cerastoderma edule*	[52,54] *
	*Solen grandis*	[39]
	*Panope japonica*	[39]
	*Patinopecten yessoensis*	[39]
	*Perna viridis*	[53]
	*Chalmys nobilis*	[53]
	*Mytilus galloprovincialis*	[54] *
	*Venerupis pullastra*	[54] *
	*Crassostrea japonica*	[54] *
	*Donax trunculus*	[54] *
	*Chamelea gallina*	[54] *
	*Gram-negative bacteria* (isolated from *C. edule*)	[55]
	Humans	[56]
Sulfocarbamoylase	*Peronidia venulosa*	[3,40]
	*Spisula solida*	[32]
	*Mya arenaria*	[32]
N-sulfotransferase-PST synthesis	*Gymnodinium catenatum*	[3,57,58]
	*Alexandrium catennela*	[59]
	*Alexandrium tamarense*	[60]
	*Cylindrospermopsis raciborskii* T3	[63,65],
	*Raphidiopsis brookii* D9	[63]
	*Anabaena circinalis*	[67,68]
	*Aphanizomenon* sp. *Nostocales*	[67,68]
	*Scytonema crispum*	[68]
	*Microseira wollei*	[64]
O-sulfotransferase-PST synthesis	*Gymnodinium catenatum*	[57,58,61]
	*Cylindrospermopsis raciborskii* T3	[63,65],
	*Raphidiopsis brookii* D9	[63]
	*Anabaena circinalis*	[67,68]
	*Aphanizomenon* sp. *Nostocales*	[66,67]
	*Scytonema crispum*	[68]
	*Microseira wollei*	[64]
N-sulfotransferase-PST metabolism	*Mytilus edulis*	[29]
	*Plactopecten magellanicus*	[37]
	*Panopea globosa*	[43]
	*Chlamys farreri*	[71,72,73]
	*Mytilus galloprovincialis*	[72,73]
	*Patinopecten yessoensis*	[74]
	*Saxidomus purpuratus*	[74]
	*Pseudomonas* sp. and *Vibrio* sp. (isolated from *A. floridus* and *T. argyrostoma*)	[75,76]
Rieske oxygenases	*Microseira wollei*	[31,69]
Reduction at N1 position	*Placopecten magellanicus*	[37]
	*Pseudocardium sachalinensis*	[80]
	*Hiatula rostrata*	[81]
	*Chlamys farreri*	[82]
	Bacteria (isolated from *M. edulis* and *E. arcuatus*)	[55]
	*Pseudomonas* sp. and *Vibrio* sp. (isolated from *A. floridus* and *T. argyrostoma*)	[76]
	α- and γ-Proteobacteria (isolated from dinoflagellates)	[83]
Oxidation at N1 position	*Alexandrium tamarense*	[3]
	*Cylindrospermopsis raciborskii* T3	[64,65,66]
	*Aphanizomenon flosaquae* NH-5	[64,65,66]
	*Microseira wollei*	[64,65,66]
	*Ahrensia* sp. and *Caulobacter* sp. (isolated from *dinoflagellates*)	[83]
	Humans	[56,85,86]
Glutathione-S-transferase	*Salmo salar*	[88]
	*Diplodus sargus*	[89,90]
Glucuronosyltransferases	Humans	[84,85]

* Conversion of benzoate paralytic shellfish toxins (PSTs).
